# Contribution of mutational resistance mechanisms and acquired β-lactamases to cefiderocol/xeruborbactam susceptibility in *Pseudomonas aeruginosa*

**DOI:** 10.1128/aac.01048-25

**Published:** 2025-10-21

**Authors:** Lucía González-Pinto, María Antonia Gomis-Font, Gloria Pérez-Rodríguez, Tania Blanco-Martín, Salud Rodríguez-Pallares, Lucía Sánchez-Peña, Alejandro Beceiro, Germán Bou, Katy Jeannot, Antonio Oliver, Jorge Arca-Suárez

**Affiliations:** 1 Servicio de Microbiología Clínica & Grupo de Investigación en Microbiología, Instituto de Investigación Biomédica de A Coruña (INIBIC), Complexo Hospitalario Universitario de A Coruña (CHUAC), Sergas, Universidade da Coruña (UDC) 16811, A Coruña, Spain; 2 CIBER de Enfermedades Infecciosas (CIBERINFEC), Instituto de Salud Carlos III 38176, Madrid, Spain; 3 Servicio de Microbiología, Servicio de Microbiología and Unidad de Investigación, Hospital Universitario Son Espases, Instituto de Investigación Sanitaria Illes Balears (IdISBa) 219656, Palma de Mallorca, Spain; 4 Departamento de Fisioterapia, Medicina y Ciencias Biomédicas, Universidade da Coruña (UDC) 16737, A Coruña, Spain; 5 Laboratoire de Bactériologie, Centre Hospitalier Universitaire de Besançon 55049, Besançon, France; 6 Laboratoire associé au Centre National de Référence de la Résistance aux Antibiotiques, Centre Hospitalier Universitaire de Besançon 55049, Besançon, France; 7 Laboratoire Chrono-Environnement UMR 6249, CNRS, Université de Franche-Comté 27000 , Besançon, France; University of Fribourg, Fribourg, Switzerland

**Keywords:** *Pseudomonas aeruginosa*, β-lactam resistance, β-lactamase, mutational mechanisms, cefiderocol, xeruborbactam, taniborbactam, β-lactamase inhibitor, β-lactam

## Abstract

Cefiderocol/xeruborbactam is a novel β-lactam/β-lactamase inhibitor combination in which the siderophore-cephalosporin cefiderocol is paired with xeruborbactam, a broad-spectrum inhibitor that targets class A to D β-lactamases. We evaluated the contribution of *Pseudomonas aeruginosa* resistance mechanisms to cefiderocol/xeruborbactam susceptibility. A panel of 61 *P*. *aeruginosa* PAO1 derivatives was tested, including 20 knockout mutants representing key chromosomal resistance mechanisms (e.g., *ampC* overexpression, efflux upregulation, porin loss, iron uptake deficiency) and 41 transformants producing major circulating β-lactamases. Xeruborbactam was assessed in combination with cefiderocol and cefepime at 4 and 8 mg/L and compared with taniborbactam. Additionally, 99 cefiderocol-resistant clinical *P. aeruginosa* isolates were evaluated. Cefiderocol/xeruborbactam retained activity against most *P. aeruginosa* mutants with chromosomally encoded resistance mechanisms. However, the *P. aeruginosa piuC-*defective mutant yielded increased cefiderocol minimum inhibitory concentrations (MIC = 2 mg/L), which could not be restored by xeruborbactam. Xeruborbactam significantly increased the activity of cefiderocol against the majority of *P. aeruginosa* PAO1 transformants, including those producing PER-1, SHV-12, KPC Ω-loop mutants, or NDM variants. Cefiderocol/xeruborbactam was active against IMP-type MBLs (which only weakly hydrolyze cefiderocol), including xeruborbactam-resistant enzymes. Relative to taniborbactam, xeruborbactam-based combinations showed similar activity against *P. aeruginosa* PAO1 transformants, but with slightly higher MIC values when tested against metallo-β-lactamase producers. This MIC increase in xeruborbactam-based combinations was partly because of the constitutive MexAB-OprM efflux in the *P. aeruginosa* background, as confirmed with *P. aeruginosa* PAO1 efflux mutants. Importantly, xeruborbactam restored susceptibility in 78% of 99 cefiderocol-resistant *P. aeruginosa* strains, reducing the MIC_90_ from 64 to 4 mg/L. Cefiderocol/xeruborbactam shows promising activity against *P. aeruginosa*.

## INTRODUCTION

*Pseudomonas aeruginosa* is a high-priority pathogen frequently implicated in life-threatening infections, particularly in critically ill or immunocompromised patients. Severe *P. aeruginosa* infections are often associated with significant morbidity and mortality, largely due to limited treatment options resulting from high levels of resistance to broad-spectrum β-lactams, including cephalosporins and carbapenems (1). β-lactam resistance in *P. aeruginosa* is typically driven by the accumulation of multiple resistance mechanisms within a single strain, leading to so-called “difficult-to-treat” phenotypes (2). These mechanisms commonly involve mutations that upregulate the expression of *ampC* and efflux pump operons, as well as disruptions of the outer membrane porin OprD. In recent years, the situation has been further exacerbated by the horizontal acquisition of genes encoding β-lactam resistance mechanisms, such as extended-spectrum β-lactamases (ESBLs) and carbapenemases (3). These enzymes are increasingly disseminated worldwide, particularly among epidemic high-risk clones such as ST235, which are particularly prone to acquiring mobile genetic elements conferring broad-spectrum resistance to β-lactams (4). The recent introduction of novel β-lactam/β-lactamase inhibitor combinations, such as ceftolozane/tazobactam, ceftazidime/avibactam, and imipenem/relebactam, has helped to partly mitigate the clinical impact of β-lactam-resistant *P. aeruginosa* infections (5). However, these agents are not active against metallo-β-lactamase (MBL)-producing strains, the most prevalent carbapenemases in *P. aeruginosa* strains worldwide (6). In addition, resistance to these combinations can emerge through mutational events that are selected during therapy, such as structural modifications of AmpC (ceftolozane/tazobactam and ceftazidime/avibactam) (7) or the combination of OprD loss with overproduction of the MexAB-OprM efflux pump (imipenem/relebactam) (8). In this context, the antipseudomonal arsenal must be urgently expanded.

Cefiderocol is a recently FDA-approved β-lactam antibiotic with potent antipseudomonal activity. Its chemical structure incorporates key elements from earlier cephalosporins: the carboxypropanoxyimino group of ceftazidime or ceftolozane at the R1 position and the pyrrolidinium group of cefepime at the C3 position, which confer increased stability against β-lactamases and enhance the affinity for penicillin-binding proteins (PBPs) (9). However, the most distinctive feature of cefiderocol is the presence of a chlorocatechol moiety at the R2 position, which enables the molecule to chelate free ferric iron (Fe³^+^) and also access the periplasm via siderophore-mediated iron uptake pathways. This mechanism allows cefiderocol to reach significantly higher periplasmic concentrations than those of conventional β-lactams. These combined structural features result in potent *in vitro* activity against a wide range of priority Gram-negative pathogens, including *P. aeruginosa*, regardless of the underlying resistance mechanisms (10). Nevertheless, the growing prevalence of acquired β-lactamases with potent cephalosporinase activity, such as NDM-1 and PER-1, has increasingly been associated with cefiderocol resistance in *P. aeruginosa* (10). In addition, studies using transposon mutant libraries and large collections of surveillance and laboratory isolates have shown that the functional loss of genes involved in iron uptake, such as *pirA*, *pirR*, *piuA*, and *piuC*, represents another major mechanism driving cefiderocol resistance in *P. aeruginosa* strains (11–13). These resistance mechanisms compromise the efficacy of cefiderocol, limiting its potential as one of the most promising antipseudomonal agents developed in recent years.

In an effort to develop strategies to protect and enhance the activity of cefiderocol, the siderophore-cephalosporin has recently entered clinical trial evaluation in combination with xeruborbactam, a novel, broad-spectrum boronate-type β-lactamase inhibitor developed through strategic modification of a benzoxaborinine scaffold (ClinicalTrials.gov ID: NCT06547554) (14). Formerly designated QPX7728, xeruborbactam has demonstrated potent inhibitory activity against class A, B, C, and D β-lactamases, with inhibition observed in the nanomolar range (15). Notably, its inhibitory spectrum surpasses that of other recently developed boronate inhibitors, such as taniborbactam, as it also targets IMP-type MBLs. To date, the activity of xeruborbactam has primarily been assessed in combination with meropenem, showing high potency against multidrug-resistant Gram-negative pathogens, including strains resistant to recently approved β-lactam/β-lactamase inhibitor combinations (16). However, our recent findings demonstrated that xeruborbactam has limited ability to potentiate meropenem activity against *P. aeruginosa*, likely due to the prevalence of β-lactamase-independent carbapenem resistance mechanisms in this species (17). Nonetheless, replacing meropenem with cefiderocol as a partner for xeruborbactam may provide meaningful therapeutic advantages, particularly for the treatment of *P. aeruginosa* infections. To our knowledge, however, the ability of the cefiderocol/xeruborbactam combination to overcome the highly complex β-lactam resistance mechanisms in *P. aeruginosa*, whether mutational or acquired via horizontal gene transfer, and especially those compromising cefiderocol efficacy, has not yet been explored.

Thus, to contribute to the therapeutic positioning of cefiderocol/xeruborbactam against *P. aeruginosa* and report on its clinical development, we sought here to evaluate the *in vitro* activity and stability of the combination against the main β-lactam resistance mechanisms in *P. aeruginosa*, including those affecting cefiderocol, and to compare its potency with that of other recently developed β-lactamase inhibitors in the *P. aeruginosa* background.

## RESULTS AND DISCUSSION

### Impact of chromosomally encoded resistance mechanisms on susceptibility to cefiderocol/xeruborbactam in *P. aeruginosa*

The minimum inhibitory concentrations (MICs) of cefiderocol, cefiderocol/xeruborbactam, and other relevant antipseudomonal comparators tested against key *P. aeruginosa* mutants with chromosomally encoded β-lactam resistance mechanisms are shown in Table 1. The relative inhibitory activity of xeruborbactam and taniborbactam against these strains is also presented, with both cefepime and cefiderocol as β-lactam reporters. The impact of adding xeruborbactam (at 4 or 8 mg/L) on the MIC of either cefepime or cefiderocol was also considered. Cefiderocol exhibited remarkable activity against this set of isolates, underscoring its status as the most potent antipseudomonal agent currently available. As previously reported, cefiderocol MICs for all tested mutants remained within the wild-type range, comparable to those of the parental *P. aeruginosa* PAO1 strain, with consistently low values between 0.06 and 0.125 mg/L. These results further support the ability of cefiderocol to overcome classical β-lactam resistance mechanisms, such as *ampC* overexpression, efflux pump upregulation, and *oprD* inactivation, even when these mechanisms are present in combination (17). The exceptionally high intrinsic activity leaves little room for xeruborbactam to further enhance the activity of cefiderocol, as evidenced by the identical MIC values obtained for cefiderocol alone and in combination with xeruborbactam (at both 4 and 8 mg/L) across all strains tested. The only mutant in the collection that yielded a meaningful increase in resistance to cefiderocol and the combination was that carrying a *piuC* inactivation. Loss-of-function mutations in *piuC* are known to be strongly selected under cefiderocol pressure, as previously described both *in vitro* and *in vivo* (13, 18). In our *piuC* mutant, cefiderocol MICs increased by 16- to 32-fold (MIC = 2 mg/L), indicating that depriving *P. aeruginosa* of PiuC-mediated uptake reduces the activity of cefiderocol to levels comparable to those of conventional cephalosporins, such as ceftazidime or cefepime, for which MICs of 1–2 mg/L are typically observed in strains lacking acquired resistance mechanisms. Thus, these findings demonstrate that the potentiating effect of xeruborbactam will be compromised in strains in which cefiderocol MICs are increased due to impaired iron uptake. MIC testing of all mutants carrying chromosomally encoded resistance mechanisms did not reveal significant differences in cefiderocol MICs when taniborbactam or xeruborbactam were used as inhibitors.

**TABLE 1:** Antibiotic susceptibility data for *P. aeruginosa* PAO1-derived mutants with the most relevant *P. aeruginosa* mutation-driven β-lactam resistance mechanisms

Strain	Genotype	Phenotype	MIC (mg/L)^*a*^^,^^*b*^													
			CAZ (R > 8)	C/A (R > 8)	C/T (R > 4)	ATM (R > 16)	FEP (R > 8)	F/TAN (4 mg/L)^*c*^ (R > 8)	F/XER (4 mg/L)^*c*^ (R > 8)	F/XER (8 mg/L)^*c*^ (R > 8)	FDC (R > 2)	FDC/TAN (4 mg/L)^*c*^ (R > 2)	FDC/XER (4 mg/L)^*c*^ (R > 2)	FDC/XER (8 mg/L)^*c*^ (R > 2)	IMP (R > 4)	MEM (R > 8)
PAO1	Wild-type	Wild-type	≤1	≤1	≤0.5	≤4	2	2	2	2	0.125	0.125	0.125	0.125	1	0.5
PAO1 Δ*ampC*	*ampC* knockout mutant	AmpC deficiency	≤1	≤1	≤0.5	≤4	2	2	2	2	0.125	0.125	0.125	0.125	0.25	0.25
PAO1 Δ*ampD*	*ampD* knockout mutant	*ampC* overexpression [↑*ampC* ≈ 50-fold]	8	2	1	≤4	8	2	4	2	0.125	0.125	0.125	0.125	1	1
PAO1 Δ*ampD* Δ*ampDh2* Δ*ampDh3*	*ampD-ampDh2-ampDh3* knockout mutant	*ampC* overexpression [↑*ampC* ≈ 1,000-fold]	32	2	2	8	16	2	4	2	0.125	0.125	0.125	0.125	1	0.5
PAO1 Δ*dacB*	*dacB* (PBP4) knockout mutant	*ampC* overexpression [↑*ampC* ≈ 50-fold]	16	2	1	8	16	2	2	2	0.125	0.125	0.125	0.125	1	1
PAO1 Δ*dacB* Δ*ampD*	*dacB* (PBP4)-*ampD* knockout mutant	*ampC* overexpression [↑*ampC* ≈ 1,500-fold]	64	4	2	16	32	2	16	8	0.125	0.125	0.125	0.125	1	1
PAO1 Δ*dacB* Δ*dacC*	Dual *dacB-dacC* (PBP4-PBP5) knockout mutant	*ampC* overexpression [↑*ampC* ≈ 500-fold]	16	≤1	2	8	16	2	8	4	0.125	0.125	0.06	0.06	0.5	≤0.125
PAO1 Δ*dacB* Δ*dacC* Δ*pbpG*	Triple *dacB-dacC-pbpG* (PBP4-PBP5-PBP7) knockout mutant	*ampC* overexpression [↑*ampC* ≈ 1,200-fold]	16	2	2	16	8	2	4	2	0.125	0.125	0.125	0.125	0.5	0.25
PAO1 Δ*mexR*	*mexR* knockout mutant	*mexAB-oprM* overexpression [↑*mexB* ≈ 10-fold]	8	4	≤0.5	16	8	8	8	8	0.125	0.125	0.125	0.125	1	2
PAO1 Δ*ampD* Δ*mexR*	*ampD-mexR* knockout mutant	*ampC* overexpression [↑*ampC* ≈ 50-fold] + *mexAB*-oprM overexpression [↑*mexB* ≈ 10-fold]	32	4	2	16	16	8	16	8	0.125	0.125	0.125	0.125	1	2
PAO1 Δ*mexZ*	*mexZ* knockout mutant	*mexXY* overexpression [↑*mexY* ≈ 15-fold]	2	≤1	1	≤4	8	8	8	8	0.125	0.125	0.125	0.125	1	0.25
PAO1 Δ*nfxB*	*nfxB* knockout mutant	*mexCD-oprJ* overexpression [↑*mexD* ≈ 150-fold]	≤1	2	1	≤4	8	8	8	8	0.125	0.125	0.125	0.125	1	0.25
PAO1 ∆*oprM*	*oprM* knockout mutant	Impaired MexAB-oprM and MexXY activity	≤1	≤1	≤0.5	≤4	0.25	0.125	0.25	0.125	0.06	0.06	≤0.03	≤0.03	1	0.5
PAO1 Δ*piuC*	*piuC* knockout mutant	Impaired iron uptake	≤1	≤1	≤0.5	≤4	2	2	2	2	2	2	2	2	1	0.5
PAOD1	PAO1 *oprD* spontaneous mutant	OprD deficiency	≤1	≤1	≤0.5	≤4	2	2	2	2	0.125	0.125	0.125	0.125	8	4
PAOD1 Δ*ampC*	Dual *ampC* knockout mutant-*oprD* spontaneous mutant	AmpC deficiency + OprD deficiency	≤1	≤1	≤0.5	≤4	2	2	2	2	0.125	0.125	0.125	0.125	0.25	2
PAOD1 Δ*ampD*	Dual *ampD* knockout mutant-*oprD* spontaneous mutant	*ampC* overexpression [↑*ampC* ≈ 50-fold] + OprD deficiency	16	≤1	1	8	8	2	4	2	0.125	0.125	0.125	0.125	16	4
PAOD1 Δ*dacB*	Dual *dacB* knockout mutant-*oprD* spontaneous mutant	*ampC* overexpression [↑*ampC* ≈ 50-fold] + OprD deficiency	32	2	2	8	16	2	4	2	0.125	0.125	0.125	0.125	8	4
PAOD1 Δ*mexR*	Dual *mexR* knockout mutant-*oprD* spontaneous mutant	*mexAB-oprM* overexpression [↑*mexB* ≈ 10-fold] + OprD deficiency	8	2	1	16	8	8	8	8	0.125	0.125	0.125	0.125	16	32
PAOD1 Δ*mexZ*	Dual *mexZ* knockout mutant-*oprD* spontaneous mutant	*mexXY* overexpression [↑*mexY* ≈ 15-fold] + OprD deficiency	≤1	≤1	1	≤4	8	8	8	8	0.125	0.125	0.125	0.125	16	4
PAOD1 Δ*dacB* Δ*mexR*	Triple *dacB* knockout mutant*-mexR* knockout mutant-*oprD* spontaneous mutant	*ampC* overexpression [↑*ampC* ≈ 50-fold] + + *mexAB-oprM* overexpression [↑*mexB* ≈ 10-fold] + OprD deficiency	16	8	1	32	16	8	8	8	0.125	0.125	0.125	0.125	8	16

^*a*^ CAZ, ceftazidime; C/A, ceftazidime/avibactam; C/T, ceftolozane/tazobactam; ATM, aztreonam; FEP, cefepime; F/TAN, cefepime/taniborbactam; F/XER, cefepime/xeruborbactam; FDC, cefiderocol; FDC/TAN, cefiderocol/taniborbactam; FDC/XER, cefiderocol/xeruborbactam; IMP, imipenem; MEM, meropenem.

^*b*^ EUCAST v.15.0 breakpoints for *Pseudomonas* spp. indicated. For combinations not yet approved, the breakpoint of the β-lactam alone is indicated.

^*c*^ Concentration at which the β-lactamase inhibitor was added.

On the other hand, using cefepime as a reporter enabled us to better analyze the enhancing effect of xeruborbactam against strains with these mutation-driven resistance mechanisms and to compare its potency with that of taniborbactam. Both taniborbactam and xeruborbactam reduced the MIC of cefepime against all mutants showing *ampC* overexpression. This adds further evidence and aligns with data from other authors about the potent coverage by both taniborbactam and xeruborbactam against the PDC β-lactamase, by far the most important cephalosporin resistance mechanism in *P. aeruginosa* (19, 20). The effect of adding xeruborbactam at 4 or 8 mg/L did not cause important changes in the MIC of cefepime, although in some cases, a concentration of 4 mg/L tended to result in non-significant slightly higher MICs (one dilution). With respect to the strains showing other mechanisms, the following were observed: (i) overproduction of MexAB-OprM, MexXY, and MexCD-OprJ caused an increase in the cefepime MICs in all cases, and the addition of either taniborbactam or xeruborbactam had a negligible effect; (ii) inactivation of OprM led to hypersusceptibility to all β-lactams, a well-characterized phenomenon attributed to the natural activity of MexAB-OprM (21); and (iii) the loss of OprD did not have any effect on the activity of non-carbapenem β-lactams.

### Impact of acquired β-lactamases on susceptibility to cefiderocol/xeruborbactam in *P. aeruginosa*

The activity of cefiderocol/xeruborbactam was evaluated against a panel of 41 *P*. *aeruginosa* PAO1 laboratory transformants producing diverse clinically relevant β-lactamases. The comparative inhibitory potency of xeruborbactam and taniborbactam was also assessed in combination with cefepime and cefiderocol (Table 2). In contrast to the isogenic isolates with mutational mechanisms, some of the β-lactamases caused a large increase in the MICs of both cefepime and cefiderocol, which enabled us to precisely determine the potency and spectrum of β-lactamase coverage of xeruborbactam. The cefiderocol/xeruborbactam combination was active against 36 of the 41 transformants included in our model, restoring cefiderocol susceptibility in 7 of 13 cefiderocol-resistant isolates and, in most cases, reducing the cefiderocol MICs to wild-type values.

**TABLE 2:** Antibiotic susceptibility data for *P. aeruginosa* PAO1-derived recombinant isolates producing β-lactamases acquired by horizontal gene transfer

Strain	Ambler class	Phenotype	MIC (mg/L)^*a*^^,^^*b*^													
			CAZ (R > 8)	C/A (R > 8)	C/T (R > 4)	ATM (R > 16)	FEP (R > 8)	F/TAN (4 mg/L)^*c*^ (R > 8)	F/XER (4 mg/L)^*c*^ (R > 8)	F/XER (8 mg/L)^*c*^ (R > 8)	FDC (R > 2)	FDC/TAN (4 mg/L)^*c*^ (R > 2)	FDC/XER (4 mg/L)^*c*^ (R > 2)	FDC/XER (8 mg/L)^*c*^ (R > 2)	IMP (R > 4)	MEM (R > 8)
PAO1	–^*d*^	Wild-type	≤1	≤1	≤0.5	≤4	2	2	2	2	0.125	0.125	0.125	0.125	1	0.5
PAO1 + GES-1	A	ESBL	32	8	16	≤4	16	2	2	2	0.25	0.125	0.125	0.125	1	0.5
PAO1 + GES-5	A	Carbapenemase	4	≤1	2	≤4	4	2	2	2	0.25	0.125	0.125	0.125	2	4
PAO1 + GES-7	A	ESBL	256	8	64	8	16	2	2	2	0.25	0.125	0.25	0.25	1	0.5
PAO1 + GES-15	A	ESBL	32	8	16	8	16	2	2	2	0.5	0.125	0.25	0.125	1	0.5
PAO1 + GES-20	A	Carbapenemase	4	≤1	4	≤4	4	2	4	2	0.25	0.125	0.125	0.25	1	8
PAO1 + CTX-M-9	A	ESBL	4	≤1	1	16	512	2	4	2	0.25	0.125	0.125	0.25	1	1
PAO1 + CTX-M-15	A	ESBL	32	≤1	2	128	512	2	4	2	0.5	0.125	0.125	0.125	1	0.5
PAO1 + SHV-12	A	ESBL	>512	16	128	>512	4,096	32	256	256	>64	1	32	4	1	2
PAO1 + PER-1	A	ESBL	512	32	>256	>512	128	2	2	2	4	0.125	0.25	0.125	1	0.5
PAO1 + VEB-1	A	ESBL	>512	32	>256	>512	256	4	4	2	4	0.125	0.25	0.125	1	0.5
PAO1 + VEB-25	A	ESBL	>512	>512	>256	>512	512	2	8	4	4	0.125	0.25	0.125	1	1
PAO1 + BEL-1	A	ESBL	16	2	8	64	16	2	2	2	0.5	0.125	0.125	0.125	1	1
PAO1 + SFO-1	A	ESBL	64	4	32	512	512	4	16	4	0.5	0.125	0.125	0.125	1	1
PAO1 + KPC-2	A	Carbapenemase	>512	4	>256	512	512	4	2	2	0.5	0.125	0.125	0.125	64	64
PAO1 + KPC-3	A	Carbapenemase	>512	4	>256	>512	512	2	2	2	1	0.125	0.125	0.125	64	64
PAO1 + KPC-31	A	ESBL	>512	512	>256	256	256	8	4	2	8	0.5	0.25	0.125	1	2
PAO1 + KPC-35	A	ESBL	>512	32	>256	64	256	4	4	2	4	0.125	0.125	0.125	1	2
PAO1 + VIM-1	B	Carbapenemase	>512	>512	>256	≤4	1,024	64	512	256	2	0.5	1	0.5	16	32
PAO1 + VIM-2	B	Carbapenemase	128	64	>256	≤4	64	2	2	2	0.5	0.125	0.125	0.125	8	16
PAO1 + VIM-20	B	Carbapenemase	64	64	>256	≤4	128	2	4	2	0.5	0.125	0.125	0.25	32	32
PAO1 + IMP-1	B	Carbapenemase	>512	>512	>256	4	512	512	512	256	0.5	0.5	0.25	0.25	8	16
PAO1 + IMP-8	B	Carbapenemase	>512	>512	>256	4	1,024	1,024	512	256	0.5	0.5	0.25	0.25	8	16
PAO1 + IMP-13	B	Carbapenemase	>512	512	>256	≤4	256	256	256	128	1	1	0.5	0.5	8	16
PAO1 + IMP-23	B	Carbapenemase	>512	>512	>256	4	1,024	1,024	1,024	1024	1	1	1	1	4	64
PAO1 + IMP-94	B	Carbapenemase	>512	>512	>256	≤4	512	512	256	128	0.5	0.5	0.25	0.5	8	64
PAO1 + NDM-1	B	Carbapenemase	>512	>512	>256	≤4	2,048	256	2,048	1,024	64	2	8	4	32	>128
PAO1 + NDM-5	B	Carbapenemase	>512	>512	>256	≤4	2,048	256	2,048	1,024	32	2	8	4	32	>128
PAO1 + NDM-7	B	Carbapenemase	>512	>512	>256	≤4	4,096	256	2,048	1,024	64	2	8	4	>128	>128
PAO1 + NDM-23	B	Carbapenemase	>512	>512	>256	≤4	2,048	256	1,024	1,024	>64	2	16	4	32	>128
PAO1 + SPM-1	B	Carbapenemase	>512	>512	>256	4	512	32	512	512	16	1	16	16	4	64
PAO1 + CMY-2	C	Extended-spectrum cephamycinase	512	4	64	128	128	8	32	16	1	0.25	0.5	0.25	2	4
PAO1 + DHA-1	C	Extended-spectrum cephamycinase	256	≤1	32	32	4	2	2	2	0.25	0.125	0.125	0.125	1	1
PAO1 + FOX-4	C	Extended-spectrum cephamycinase	256	32	16	16	16	2	2	2	0.25	0.125	0.25	0.125	1	1
PAO1 + CMH-7	C	Extended-spectrum cephamycinase	128	2	4	64	8	2	2	2	0.25	0.125	0.125	0.125	1	2
PAO1 + OXA-1	D	Narrow-spectrum oxacillinase	2	2	1	4	512	512	8	4	1	0.5	0.25	0.125	1	1
PAO1 + OXA-2	D	Narrow-spectrum oxacillinase	64	4	2	64	128	2	2	2	2	0.125	0.25	0.125	4	8
PAO1 + OXA-10	D	Narrow-spectrum oxacillinase	2	≤1	2	32	16	2	2	2	0.25	0.125	0.125	0.125	2	8
PAO1 + OXA-14	D	ESBL	512	256	128	32	128	8	32	8	4	2	2	1	2	4
PAO1 + OXA-15	D	ESBL	256	64	128	16	16	4	4	4	4	2	1	1	2	1
PAO1 + OXA-24	D	Carbapenemase	2	2	0.5	≤4	4	2	2	2	0.125	0.125	0.125	0.125	2	32
PAO1 + OXA-48	D	Carbapenemase	2	2	2	≤4	16	2	2	2	0.125	0.125	0.125	0.125	16	64

^*a*^ CAZ, ceftazidime; C/A, ceftazidime/avibactam; C/T, ceftolozane/tazobactam; ATM, aztreonam; FEP, cefepime; F/TAN, cefepime/taniborbactam; F/XER, cefepime/xeruborbactam; FDC, cefiderocol; FDC/TAN, cefiderocol/taniborbactam; FDC/XER, cefiderocol/xeruborbactam; IMP, imipenem; MEM, meropenem.

^*b*^ EUCAST v.15.0 breakpoints for *Pseudomonas* spp. indicated. For combinations not yet approved, the breakpoint of the β-lactam alone is indicated.

^*c*^ Concentration at which the β-lactamase inhibitor was added.

^*d*^ –, no data.

Regarding class A enzymes, cefiderocol alone retained activity against many transformants producing β-lactamase variants known to weakly inactivate this antibiotic, such as GES- and CTX-M-like enzymes. However, the MICs increased to levels above the EUCAST clinical breakpoint in the transformants producing SHV-12 (MIC >64 mg/L), PER-1 (MIC = 4 mg/L), VEB-1 (MIC = 4 mg/L), VEB-25 (MIC = 4 mg/L), and the KPC Ω-loop variants KPC-31 (MIC = 8 mg/L) and KPC-35 (MIC = 4 mg/L), all previously reported as common drivers of cefiderocol resistance (22–24). The addition of xeruborbactam decreased the cefiderocol MIC for almost all transformants to wild-type levels, except for the transformant producing SHV-12, which yielded MICs of 32 mg/L (with xeruborbactam at 4 mg/L) and 4 mg/L (with xeruborbactam at 8 mg/L). Although SHV-12 has previously been associated with resistance to cefiderocol in laboratory isolates of *P. aeruginosa* and Enterobacterales, as well as in clinical strains of *Enterobacter cloacae* complex (25), its distribution is very limited in clinical strains of *P. aeruginosa*. Thus, SHV-12 is not expected to be a prominent mechanism of cefiderocol/xeruborbactam resistance in *P. aeruginosa* in the near future.

With respect to transformants producing class B β-lactamases, cefiderocol/xeruborbactam demonstrated some activity, although the MICs varied, depending on the specific enzymatic variant. The combination remained active against the transformants producing VIM-1, VIM-2, VIM-20, and IMP-type enzymes, with MICs ranging from 0.125 to 1 mg/L, and with no significant differences observed between the 4 and 8 mg/L concentrations of xeruborbactam. This activity is partly explained by the intrinsic stability of cefiderocol against these enzymes, as the MICs of the β-lactam alone ranged between 0.125 and 2 mg/L. Compared to transformants producing enzymes from other Ambler classes included in our study, it is worth noting that, although xeruborbactam has been shown to increase the activity of β-lactams against isogenic Enterobacterales strains producing MBLs (26), its enhancing effect is less pronounced against class B enzymes produced in *P. aeruginosa*, as recently demonstrated by Le Terrier *et al*. (27). The reduced potency likely stems from multiple *P. aeruginosa*-specific factors: (i) limited outer membrane permeability hindering uptake of the inhibitor; (ii) decreased intrinsic activity against *P. aeruginosa* PBPs, relative to *Escherichia coli*; (iii) and active efflux via MexAB-OprM, reducing periplasmic accumulation of xeruborbactam (28).

Interestingly, our isogenic panel included a *P. aeruginosa* PAO1 transformant producing IMP-23, which harbors a V67F amino acid substitution previously shown to sterically hinder the positioning of xeruborbactam within the active site, thereby impairing its inhibitory effect. This type of substitution, already present in circulating IMP variants, could represent an emerging threat to the efficacy of xeruborbactam, as reported in earlier studies evaluating its activity in combination with meropenem (29). However, replacing meropenem with cefiderocol, an antibiotic poorly hydrolyzed by IMP enzymes, appears to mitigate the negative impact of such substitutions on the efficacy of the combination. Nevertheless, the potential emergence of similar substitutions in other class B enzymes with higher intrinsic activity against cefiderocol, such as NDM variants, warrants close surveillance upon clinical introduction. Regarding NDM enzymes, which, due to their global spread and potent cephalosporinase activity, represent one of the most serious threats to cefiderocol (30), xeruborbactam consistently reduced MICs from 32->64 mg/L to 4 mg/L (a 16-fold decrease) in all NDM-producing transformants, indicating its potential to restore cefiderocol activity. Although an MIC of 4 mg/L remains above current susceptibility breakpoints, this reflects the elevated baseline MICs in our overexpression model. Thus, resistance to the cefiderocol/xeruborbactam combination is unlikely in clinical *P. aeruginosa* NDM-1 strains that lack additional resistance mechanisms, as most circulating NDM-1-producing *P. aeruginosa* isolates typically yield cefiderocol MICs between 2 and 16 mg/L (31). Finally, the SPM-1-producing transformant, carrying one of the most prevalent MBLs in South America, yielded a cefiderocol MIC of 16 mg/L, which remained unaffected by the addition of xeruborbactam, highlighting a potential limitation of the combination in this context.

With respect to the transformants producing class C and class D β-lactamases, both cefiderocol and cefiderocol/xeruborbactam exhibited potent activity. The addition of xeruborbactam tended to reduce cefiderocol MICs, when elevated, back to wild-type levels (0.125 mg/L). Notably, xeruborbactam was able to moderately reduce cefiderocol MICs in transformants producing the extended-spectrum oxacillinases OXA-14 and OXA-15, from 4 mg/L to 1 mg/L. Although these OXA variants are not highly prevalent, a recent study by Mack *et al*. highlighted that their respective parental enzymes, OXA-10 and OXA-2, are among the most frequently detected β-lactamases in *P. aeruginosa* genomes available in GenBank (32). Specific amino acid substitutions in these enzymes, selected during cephalosporin treatment, are associated with an extended-spectrum resistance phenotype, conferring resistance to ceftazidime/avibactam, ceftolozane/tazobactam, and cefiderocol (33, 34). Therefore, xeruborbactam may provide additional protection for cefiderocol and offer a valuable therapeutic option against these highly cephalosporin-resistant *P. aeruginosa* strains.

Regarding the relative inhibitory activity of xeruborbactam versus taniborbactam with cefepime and cefiderocol as reporters, both showed similar potency, although taniborbactam combinations were slightly more active against strains producing certain β-lactamase targets. Regarding class A enzymes, the greatest differences in potency were noted for the transformant producing SHV-12, which was more strongly inhibited by taniborbactam, with MIC values of 32 mg/L for cefepime/taniborbactam and 1 mg/L for cefiderocol/taniborbactam, compared to 256 mg/L for cefepime/xeruborbactam and 4 mg/L for cefiderocol/xeruborbactam. With respect to MBLs, taniborbactam proved to be more potent against the transformants producing VIM-1, NDM-1, and SPM-1. Considering that the inhibition kinetics for xeruborbactam against purified MBLs are, with few exceptions, better than those reported for taniborbactam (15, 20, 35), the comparative MIC values add further evidence of the impact of the *P. aeruginosa* background on the activity of this promising inhibitor. Interestingly, the transformant producing the class D β-lactamase OXA-1 was much more strongly inhibited by xeruborbactam-based combinations, with MICs of 4 mg/L for cefepime/xeruborbactam and 0.125 mg/L for cefiderocol/xeruborbactam, compared to 512 mg/L for cefepime/taniborbactam and 0.5 mg/L for cefiderocol/taniborbactam.

### Activity of xeruborbactam and novel β-lactamase inhibitors against *P. aeruginosa* efflux mutants

Given the intrinsic activity of xeruborbactam against Enterobacterales and previous reports highlighting the role of MexAB-OprM efflux in limiting its efficacy, we investigated the impact of both disruption and hyperproduction of the major resistance-nodulation-division (RND)-type efflux systems in the *P. aeruginosa* PAO1 background on xeruborbactam activity. In parallel, we compared its performance with that of other recently approved or investigational β-lactamase inhibitors (Table 3). In the wild-type *P. aeruginosa* PAO1 background, zidebactam displayed the greatest intrinsic activity (MIC = 2 mg/L), followed at a considerable distance by nacubactam (MIC = 128 mg/L), and by all remaining inhibitors (MICs >256 mg/L). Disruption of *oprM*, which encodes the outer membrane channel shared by both MexAB-OprM and MexXY, enabled us to assess the contribution of these efflux systems to the baseline susceptibility profile of *P. aeruginosa* PAO1 toward these inhibitors. Interestingly, the avibactam, nacubactam, and zidebactam MICs were only modestly reduced (twofold), while no change was observed for relebactam, vaborbactam, or taniborbactam, underscoring the lack of relevant intrinsic antibacterial activity of these agents against *P. aeruginosa*. By contrast, xeruborbactam showed the most pronounced reduction in MIC, decreasing from >256 mg/L to 64 mg/L (over fourfold) upon *oprM* deletion. These findings provide unequivocal evidence that, despite its broad-spectrum inhibitory activity against β-lactamases, xeruborbactam faces notable limitations in *P. aeruginosa*, at least partly due to active drug extrusion via RND-type efflux pumps.

**TABLE 3:** Minimum inhibitory concentrations of the β-lactamase inhibitors against the wild-type *P. aeruginosa* PAO1 strain and efflux mutants

Strain	Genotype	Phenotype	MIC (mg/L)						
			Avibactam	Relebactam	Nacubactam	Zidebactam	Vaborbactam	Taniborbactam	Xeruborbactam
PAO1	Wild-type	Wild-type	>256	>256	128	2	>256	>256	>256
PAO1 *∆mexR*	*mexR* knockout mutant	*mexAB-oprM* overexpression [↑*mexB* ≈ 10-fold]	>256	>256	128	8	>256	>256	>256
PAO1 *∆mexZ*	*mexZ* knockout mutant	*mexXY* overexpression [↑*mexY* ≈ 15-fold]	>256	>256	128	2	>256	>256	>256
PAO1 *∆nfxB*	*nfxB* knockout mutant	*mexCD-oprJ* overexpression [↑*mexD* ≈ 150-fold]	>256	>256	128	2	>256	>256	>256
PAO1 ∆*oprM*	*oprM* knockout mutant	Impaired MexAB-oprM and MexXY activity	128	>256	64	1	>256	>256	64

### Activity of cefiderocol/xeruborbactam against clinical cefiderocol-resistant *P. aeruginosa* strains and associated resistance mechanisms

To further assess the ability of xeruborbactam to potentiate the activity of cefiderocol in clinical *P. aeruginosa* isolates with acquired cefiderocol resistance mechanisms, we evaluated the efficacy of the combination against a collection of 99 representative cefiderocol-resistant isolates. The cumulative MIC distributions of cefiderocol and cefiderocol/xeruborbactam for these strains are presented in Table 4. All isolates yielded cefiderocol MICs >2 mg/L, with values ranging from 4 to >128 mg/L. The MIC_50_ and MIC_90_ were 8 mg/L and 64 mg/L, respectively. The addition of xeruborbactam significantly enhanced cefiderocol activity across the collection (*P* < 0.0001). Using a tentative breakpoint of 2 mg/L (equivalent to that of cefiderocol alone), cefiderocol/xeruborbactam restored susceptibility in 78% (77/99) of the isolates. Importantly, no isolate exhibited a MIC greater than 16 mg/L for the combination. Xeruborbactam decreased both the MIC_50_ and MIC_90_ values of cefiderocol by 16-fold, resulting in final values of 0.5 and 4 mg/L, respectively. These data support the strong potentiating effect of xeruborbactam and its potential to overcome acquired cefiderocol resistance in a substantial proportion of *P. aeruginosa* clinical isolates.

**TABLE 4:** Cumulative MIC distribution, MIC_50_, and MIC_90_ values of cefiderocol and cefiderocol/xeruborbactam against cefiderocol-resistant *P. aeruginosa* clinical isolates

		MIC (mg/L)^*a*^^,*b*^														MIC_50_	MIC_90_	MIC range	% Susc.	% Res.
		0.03	0.06	0.125	0.25	0.5	1	2	4	8	16	32	64	128	>128					
FDC-resistant Isolates (*n* = 99)	FDC								35 (35.4%)	61 (61.6%)	85 (85.9%)	87 (87.9%)	90 (90.9%)	92 (92.9%)	99 (100.0%)	8	64	4->128	0%	100%
	FDC/XER^*c*^	2 (2.2%)	5 (5.1%)	15 (15.2%)	28 (28.3%)	51 (51.5%)	66 (66.7%)	77 (77.8%)	93 (93.9%)	97 (98.0%)	99 (100.0%)					0.5	4	0.03-16	78%	22%

^*a*^ Xeruborbactam was tested at a fixed concentration of 8 mg/L with cefiderocol.

^*b*^ FDC, cefiderocol; FDC/XER, cefiderocol/xeruborbactam; Susc., susceptible; Res., resistant.

^*c*^ EUCAST v.15.0 breakpoints for *Pseudomonas* spp. indicated. For cefiderocol/xeruborbactam, the same breakpoint was applied as for cefiderocol.

We analyzed the resistome of the six clinical isolates that yielded cefiderocol/xeruborbactam MIC values >4 mg/L (Table 5), to clarify the drivers of cefiderocol/xeruborbactam resistance in clinical *P. aeruginosa* strains. These isolates carried (i) mutations or large chromosomal deletions affecting genes coding for proteins involved in iron uptake: *piuA*, *piuC*, *pirA,* and *pirR* (4/6). Interestingly, the chromosomal deletions present in *P. aeruginosa* strains 22.10391 and 24.11402 also affected the adjacent genes *ampD* and *ampE*, thus also driving increased *ampC* expression levels, as recently reported in cefiderocol-resistant clinical *P. aeruginosa* strains (12); (ii) production of β-lactamases acquired by horizontal gene transfer with potent cephalosporinase activity (3/6; including PAC-1, NDM-1, and GES-9); and (iii) alterations affecting MexAB-OprM regulators MexR or NalD, associated with increased xeruborbactam efflux (5/6). These observations confirm the results drawn from our experimental model with *P. aeruginosa* isogenic strains on the contribution of impaired iron uptake, β-lactamases, and increased MexAB-OprM efflux to cefiderocol/xeruborbactam resistance. The combination of these mechanisms in the same strain may increase the MIC of the cefiderocol/xeruborbactam combination above 4 mg/L, limiting its activity against *P. aeruginosa*.

**TABLE 5:** Genotypic features of the six clinical strains of *P. aeruginosa* with a cefiderocol/xeruborbactam MIC >4 mg/L^*a*^

Strain	ST	MIC of FDC (mg/L)	MIC of FDC/XER (mg/L)	Proteins involved in iron transport and/or regulation						Acquired β-lactamases	MexAB-OprM regulators	Other β-lactam resistance determinants
				PiuA	PiuC	PirA	PirR	FpvB	FecA			
22.9556	ST664	>128	8	T689fs	–^*b*^	–	–	–	–	PAC-1, OXA-10	NalD (S8C)	OprD (W277X), PBP-3 (L346M, F533L), MreB (truncated)
22.10391	ST500	16	8	∆	∆	–	–	–	–	–	–	OprD (Q142X), AmpE (absent), AmpD (absent)
21.9246	ST1632	64	16	*–*	G90fs	G685D	G130fs	–	E710D	–	MexR (Q106X)	OprD (F303X), MexZ (L85P), PBP-4 (S399L, H473Y), NagE (A496T), PBP-2 (I118V), MpI (P37X), MurD (M20I), PBP-1a (A749T)
23.11286	ST357	16	8	*–*	–	–	–	–	–	NDM-1, LCR-1	NalD (F51I)	AmpDh3 (R134C), OprD (M372V), ParS (A13T)
24.11402	ST3302	16	8	∆	∆	–	–	D121H	–	–	MexR (I104X)	AmpE (absent), AmpD (absent)
24.11504	ST357	128	16	*–*	**–**	–	–	–	–	GES-9, LCR-1	NalD (F51I)	AmpDh3 (R134C), OprD (W6X), ParS (A13T)

^*a*^ ST, sequence type; FDC, cefiderocol; FDC/XER, cefiderocol/xeruborbactam (with xeruborbactam tested at a fixed concentration of 8 mg/L); fs, frameshift; X, stop codon; Δ, deletion or absence.

^*b*^ –, no data.

### Concluding remarks

Here, we provide previously unreported data on the performance of the novel combination cefiderocol/xeruborbactam against well-characterized *P. aeruginosa* laboratory strains with defined resistance mechanisms, as well as a large set of cefiderocol-resistant clinical *P. aeruginosa* isolates. Our findings highlight that the addition of xeruborbactam provides limited benefit against *P. aeruginosa* strains with classic chromosomally encoded β-lactam resistance mechanisms, against which cefiderocol alone already exhibits high activity. However, we have demonstrated here that inactivating mutations in iron uptake-related genes, such as *piuA* or *piuC,* significantly elevate cefiderocol MICs in a β-lactamase-independent manner, rendering the addition of xeruborbactam ineffective in these cases. Given that such mutations are selectively enriched under cefiderocol pressure, the role of xeruborbactam as a rescue strategy for resistance driven by iron transport loss is likely to be limited. With respect to β-lactamases, cefiderocol/xeruborbactam showed broad-spectrum activity across all transformants producing enzymes from all Ambler classes and displayed comparable potency to cefiderocol partnered with taniborbactam. The activity of cefiderocol/xeruborbactam against *P. aeruginosa* strains producing MBLs may be slightly lower than against those producing other β-lactamase classes, an effect that can probably be attributed to the following: (i) lower inhibitory activity of xeruborbactam against MBLs compared to serine-type enzymes (15); (ii) the complex genetic background of *P. aeruginosa*, where efflux systems, particularly MexAB-OprM, play a major role in reducing intracellular concentrations of xeruborbactam. Nevertheless, the high intrinsic stability of cefiderocol against most MBLs, including xeruborbactam-resistant variants such as the V67F-substituted IMP-23, supports the overall effectiveness of the combination against *P. aeruginosa*. Finally, our data show that the addition of xeruborbactam restored cefiderocol susceptibility in approximately 80% of cefiderocol-resistant clinical isolates, making the combination a promising therapeutic option to target circulating *P. aeruginosa* strains that have acquired cefiderocol resistance mechanisms. Altogether, our findings provide a critical baseline of *in vitro* data to better understand the activity and mechanisms of resistance associated with the cefiderocol/xeruborbactam combination in *P. aeruginosa*. The combination emerges as a promising therapeutic option for tackling emerging cefiderocol-resistant strains, supporting its continued clinical development.

## MATERIALS AND METHODS

### Collection of *P. aeruginosa* PAO1-derived laboratory mutants with chromosomally encoded mechanisms of β-lactam resistance

We evaluated a collection comprising the *P. aeruginosa* PAO1 reference strain and 20 isogenic *P. aeruginosa* PAO1-derived mutants representing the main chromosomally encoded β-lactam resistance mechanisms in *P. aeruginosa*. This collection included the following: (i) a single knockout of the *bla*_PDC_ gene, as well as single, double, and triple knockouts of regulatory genes (*dacB*, *ampD*, *ampDh2*, *ampDh3*, *dacC*, and *pbpG*), resulting in a broad range of *bla*_PDC_ expression levels; (ii) spontaneous *oprD* mutants and knockout mutants of the local negative regulators *mexR*, *mexZ*, and *nfxB*, which respectively lead to overexpression of the major RND efflux operons *mexAB-oprM*, *mexXY*, and *mexCD-oprJ*; (iii) double or triple knockout mutants combining key resistance mechanisms; and (iv) single knockout mutants of *piuC* and *oprM*, impairing iron uptake and efflux, respectively, to investigate the roles of reduced iron acquisition in cefiderocol resistance and MexAB-OprM and MexXY efflux in β-lactam and β-lactamase inhibitor susceptibility. The recombinant isolates were constructed using the *cre-lox* gene inactivation system, and the resulting gene overexpression and/or deletion was characterized by RT-PCR or PCR followed by Sanger sequencing. Most recombinant isolates had been generated in previous studies (36–39), whereas the *P. aeruginosa* PAOD1 Δ*dacB* Δ*mexR* mutant was specifically constructed for this study using the same methodology, starting with the PAOD1 Δ*dacB* mutant. Finally, the *P. aeruginosa* PAO1 Δ*piuC* knockout mutant was also constructed using the *cre-lox* system by MA Gomis-Font as part of ongoing research.

### Collection of *P. aeruginosa* PAO1-derived laboratory transformants producing acquired broad-spectrum β-lactamases

To evaluate the specific impact of β-lactamases on susceptibility to cefiderocol/xeruborbactam and comparator agents, we used a previously engineered library of recombinant *P. aeruginosa* PAO1 strains (17). Each strain produces 1 of 41 representative β-lactamases encompassing the most clinically relevant enzymes from Ambler classes A, B, C, and D. For this work, the panel was specifically enriched with 10 additional transformants, including producers of specific inhibitor-resistant β-lactamases. To this end, the genes coding for *bla*_BEL-1_, *bla*_VEB-25_, *bla*_SFO-1_, *bla*_IMP-1_, *bla*_IMP-8_, *bla*_IMP-23_, *bla*_SPM-1_, *bla*_CMH-7_, and *bla*_OXA-24_ were amplified with specific primers, ligated to the pUCP-24 plasmid, and cloned into the *E. coli* TG1 host, as previously described. The recombinant plasmids were subsequently electroporated into the *P. aeruginosa* PAO1 strain, and transformants were selected on Luria-Bertani agar plates containing 30 mg/L gentamicin. All transformants were validated by PCR, plasmid restriction analysis, Sanger sequencing, and MIC determination.

### Cefiderocol-resistant clinical *P. aeruginosa* strains

To further investigate the potential of xeruborbactam to increase the activity of cefiderocol against *P. aeruginosa*, we evaluated its effect on a collection of 99 cefiderocol-resistant clinical isolates. This collection comprised *P. aeruginosa* strains recovered in France since 2021 and submitted to the French National Reference Center for Antibiotic Resistance in Gram-Negative Non-Fermentative Bacilli (Centre Hospitalier Universitaire de Besançon, France). All isolates exhibited confirmed cefiderocol MICs >2 mg/L, as determined by the reference broth microdilution method (described in detail below). The resistome of the isolates with cefiderocol/xeruborbactam MIC values >4 mg/L was analyzed by WGS (Illumina). Assembled genomes were explored using CARD Resistance Gene Identifier v.6.0.5 to identify β-lactamases acquired by horizontal gene transfer, while sequence variations in chromosomal genes associated with β-lactam resistance or iron uptake were assessed through variant calling using the genome of the wild-type *P. aeruginosa* PAO1 strain as reference, as previously described (40).

### Antimicrobial susceptibility testing

MICs were determined in duplicate for all isolates, with a third determination performed if results differed by more than one dilution. MIC testing was conducted with cation-adjusted Mueller-Hinton (MH) broth for all agents, except for cefiderocol, cefiderocol/taniborbactam, and cefiderocol/xeruborbactam, which were tested in iron-depleted cation-adjusted MH broth, prepared according to CLSI M100 guidelines (41), using BD-BBL brand MH broth (42). For β-lactam/β-lactamase inhibitor combinations, avibactam, tazobactam, and taniborbactam were tested at a fixed concentration of 4 mg/L. Since CLSI has not yet disclosed the xeruborbactam concentration in the cefiderocol/xeruborbactam combination, xeruborbactam was tested against laboratory isolates at both 4 mg/L and 8 mg/L in combination with cefepime (to assess the impact of inhibitor concentration and enable direct comparison with cefepime/taniborbactam) and with cefiderocol. In addition, taniborbactam was tested at a fixed concentration of 4 mg/L with cefiderocol for direct comparison with cefiderocol/xeruborbactam. For clinical isolates, cefiderocol/xeruborbactam was evaluated using a fixed xeruborbactam concentration of 8 mg/L. EUCAST v.15.0 clinical breakpoints and guidelines (http://www.eucast.org/clinical_breakpoints/) were used for reference purposes. Reference strains *E. coli* ATCC 25922, *Klebsiella pneumoniae* ATCC 700603, *E. coli* NCTC 13353, and *P. aeruginosa* ATCC 27853 were used as controls. The potency of xeruborbactam was validated in all assays by testing it in combination with meropenem against *K. pneumoniae* strain ATCC BAA-2814 (meropenem/xeruborbactam MIC range = 0.015 mg/L–0.06 mg/L), as recommended by CLSI 2025 guidelines. The intrinsic activity of avibactam, relebactam, nacubactam, zidebactam, vaborbactam, taniborbactam, and xeruborbactam was assessed by determining the MICs individually.

### Statistical analysis

Statistical analysis of the effect of xeruborbactam to increase the activity of cefiderocol against *P. aeruginosa* was performed using Fisher’s exact test with the GraphPad software.
